# β-Aminoisobutyric acid (L-BAIBA) is a novel regulator of mitochondrial biogenesis and respiratory function in human podocytes

**DOI:** 10.1038/s41598-023-27914-8

**Published:** 2023-01-14

**Authors:** Irena Audzeyenka, Maria Szrejder, Dorota Rogacka, Stefan Angielski, Moin A. Saleem, Agnieszka Piwkowska

**Affiliations:** 1 Laboratory of Molecular and Cellular Nephrology, Mossakowski Medical Research Institute, Polish Academy of Sciences, Wita Stwosza St. 63, 80-308 Gdansk, Poland; 2 Department of Molecular Biotechnology, Faculty of Chemistry, University of Gdansk, Gdansk, Poland; 3 Bristol Renal, University of Bristol, Bristol, UK

**Keywords:** Biochemistry, Cell biology, Drug discovery, Molecular biology, Kidney diseases, Metabolic disorders

## Abstract

Podocytes constitute an external layer of the glomerular filtration barrier, injury to which is a hallmark of renal disease. Mitochondrial dysfunction often accompanies podocyte damage and is associated with an increase in oxidative stress and apoptosis. β-Aminoisobutyric acid (BAIBA) belongs to natural β-amino acids and is known to exert anti-inflammatory and antioxidant effects. BAIBA has been reported to be involved in regulating mitochondrial dynamics, but unknown is whether BAIBA influences podocyte bioenergetics. The present study showed that human podocytes express the BAIBA receptor, Mas-related G protein-coupled receptor type D (MRGPRD), which is sensitive to BAIBA stimulation. The treatment of podocytes with L-BAIBA significantly increased their respiratory parameters, such as basal and maximal respiration, adenosine triphosphate (ATP) production, and spare respiratory capacity. We also found that L-BAIBA altered mitochondrial quantity, size, and shape, promoting organelle elongation and branching. L-BAIBA significantly upregulated peroxisome proliferator activated receptor γ coactivator-1α (PGC-1α) and transcription factor A mitochondrial (TFAM), indicating an increase in mitochondrial biogenesis. Our results demonstrate a novel regulatory mechanism of mitochondrial dynamics in podocytes, which may be important for maintaining their functions in the renal filtration barrier and prompting further investigations of preventing or ameliorating mitochondrial damage in podocytes in pathological states.

## Introduction

β-Aminoisobutyric acid (BAIBA) belongs to natural β-amino acids in biological systems. Two BAIBA enantiomers, L-BAIBA and D-BAIBA, are expressed in humans, each having unique metabolism processes and modes of action. The existence of D-BAIBA was originally established in 1951 after its isolation from human urine^1^. Most studies report that this enantiomer prevails in urine, whereas L-BAIBA dominates in plasma^2,3^. D-BAIBA is a catabolic product of pyrimidine base thymine. It is generated in the cytosol in a series of enzymatic reactions that involve dihydropyrimidin dehydrogenase, dihydropyrimidinase, and β-ureidopropionase and is further metabolized in mitochondria by alanine:glyoxylate aminotransferase 2 (AGXT2)^4^. L-BAIBA is a catabolic product of the branched α-amino acid L-valine. It is generated in a bidirectional reaction by the mitochondrial enzyme 4-aminobutyrate aminotransaminase from L-methylmalonate semialdehyde, a product of the enzymatic processing of L-valine^5^.

BAIBA is produced and secreted mainly by skeletal myocytes in response to exercise, which increases plasma levels of D-BAIBA and L-BAIBA by 13% and 20%, respectively^6^. Many studies demonstrated a positive impact of BAIBA supplementation on carbohydrate and lipid metabolism. BAIBA exerted beneficial effects on body composition (i.e., more lean mass and better subcutaneous/visceral adipose tissue proportion) and systolic blood pressure^7,8^. Additionally, in mouse models of type 2 diabetes (i.e., high-fat diet-induced diabetes and streptozotocin-induced diabetes), BAIBA treatment reduced fasting levels of free fatty acids, triglycerides, and low-density lipoproteins, which was associated with a decrease in hepatic lipogenesis and improvements in insulin sensitivity^9^.

Diabetic nephropathy is one of the most frequent complications of type 2 diabetes, characterized by the progressive damage of renal tissue and impairments in glomerular filtration, clinically manifested by proteinuria and hypertension. The primary hallmark of early stages of diabetic nephropathy is podocyte damage, including the flattening and effacement of podocyte foot processes, cell detachment, and apoptosis^10^. Among the mechanisms of podocyte injury in diabetes, mitochondrial dysfunction appears to play a critical role in imbalances of lipid metabolism, bioenergetics, and insulin responsiveness in podocytes^11,12^. The regulation of mitochondrial homeostasis is tightly related to their biogenesis and recycling (mitophagy) processes. Evidence suggests that one potent regulatory factor of mitochondrial dynamics is BAIBA, which induces mitochondrial biogenesis-related molecules, such as peroxisome proliferator activated receptor γ coactivator-1α (PGC-1α), PGC-1β, estrogen-related receptor α, and transcription factor A mitochondrial (TFAM) in skeletal muscle and vascular endothelial cells^13,14^. The mechanisms of signal transduction by extracellular BAIBA are still poorly understood. Several membrane receptors for BAIBA have been proposed, among which Mas-related G protein-coupled receptor type D (MRGPRD) is reported to play an important role in mitochondrial protection from reactive oxygen species-dependent injury in osteocytes^15^.

The possible effects of BAIBA on podocyte biology and renal function have not been investigated to date. Interestingly, the liver and kidney are primary sites of AGXT2 expression, a master enzyme that controls circulating levels of BAIBA^4^. Considering the established involvement of BAIBA in mitochondrial regulation in other cell types and its role in the attenuation of oxidative stress, inflammation, and insulin resistance, we hypothesized that podocytes may also be sensitive to extracellular BAIBA stimulation. The present study found that human podocytes express the BAIBA receptor MRGPRD, and L-BAIBA treatment increased mitochondrial biogenesis and podocyte respiratory function. Our findings may be important for prompting further research on possible mechanisms of podocyte protection from metabolic dysfunction in disease states, including diabetic nephropathy.

## Methods

### Cell culture and treatment

Immortalized human podocytes were provided by Prof. Moin Saleem (University of Bristol). Undifferentiated cells were cultured at 33 °C in RPMI-1640 medium (Sigma-Aldrich) that was supplemented with 10% fetal bovine serum (Gibco, Thermo Fisher) and 1% penicillin–streptomycin solution (Gibco, Thermo Fisher). To induce differentiation, cells were placed at 37 °C, and the experiments were conducted on podocytes 10–16 days afterward. L-BAIBA (Sigma-Aldrich; 10 µM) was added for the last 5 days, 2 days, or 24 h before the experiments.

### Oxygen consumption rate measurements

Measurements of oxygen consumption rates (OCRs) were performed according to a previously published protocol^11^. Briefly, podocytes were cultured in eight-well microplates (Agilent) in standard RPMI 1640 medium that was supplemented with or without 10 µM L-BAIBA (5 days, 2 days, and 24 h). Oxygen consumption rates were determined in a Seahorse XFp analyzer (Agilent) before and after an injection of 1 μM oligomycin, 1 μM carbonyl cyanide *p*-trifluromethoxyphenylhydrazone (FCCP), and 0.5 μM rotenone/antimycin. Respiration parameters and adenosine triphosphate (ATP) production were measured based on slopes of OCRs in real-time analyses and normalized to the protein concentration in each well.

### RNA/DNA isolation and real-time polymerase chain reaction

Total RNA was isolated using the RNeasy Mini Kit (Qiagen), and ~ 1500 ng of RNA was taken for cDNA synthesis. The levels of mRNA transcripts for MRGPRD, PGC-1α, TFAM, and β-actin were determined using gene-specific primers and Taq-Man hydrolysis probes. The Genomic Mini kit (A&A Biotechnology) was used for DNA isolation. DNA (~ 100 ng) was used for single real-time polymerase chain reaction (PCR) to determine the copy number of the tRNA_Leu mitochondrial gene relative to the β2-microglobulin nuclear gene (SybrGreen detection system). Real-time PCR analyses were performed in a LightCycler 480 (Roche). The results were quantified using the ΔΔCt method, with β-actin as the internal control. Sequences of primers and probes are given in Supplementary Table S1.

### Western blot

Total protein lysates (20–30 µg of protein) were separated in sodium dodecyl sulfate–polyacrylamide gel and immunoblotted on polyvinylidene difluoride membranes. Membranes were blocked in 3% bovine serum albumin and incubated with protein-specific primary antibodies. Horseradish peroxidase-conjugated secondary antibodies were used, and membranes were developed using a chemiluminescent detection system. Primary antibodies and their dilutions are given in Supplementary Table S2 and uncropped immunoblot membranes are shown in Fig. S1. Densitometric quantification of the bands was performed using ImageJ software.

### Immunofluorescent staining

The detailed immunofluorescent staining protocol and assessment of mitochondrial morphology were described previously^11^. The following primary antibodies were used: anti-MRGPRD (Biorbyt, catalog no. orb157281, 1:100) and anti-synaptopodin (Santa Cruz Biotechnology, catalog no. sc-21537; 1:50). For mitochondria staining, a 200 nM solution of MitoTracker Red (ThermoFisher) was used. Images were taken on a confocal microscope (Eclipse Ti, Nikon Instruments). The average area, perimeter, axis length, and compactness of mitochondria were determined using CellProfiler software^16^. There were 7–8 images taken into analyses per one condition, each image contained ~ 3000 segmented objects (mitochondria).

### Statistical analyses

The statistical analyses were performed using GraphPad Prism 8 (Graphpad software, USA). The Shapiro–Wilk test was used to determine normality of the datasets. For data with a normal distribution, the unpaired *t*-test was used. For other cases, the nonparametric Mann–Whitney test was used. The results are expressed as mean ± SEM. Values of *p* ≤ 0.05 were considered statistically significant.

## Results

### Human podocytes express L-BAIBA stimulation-sensitive MRGPRD

**Figure 1:**
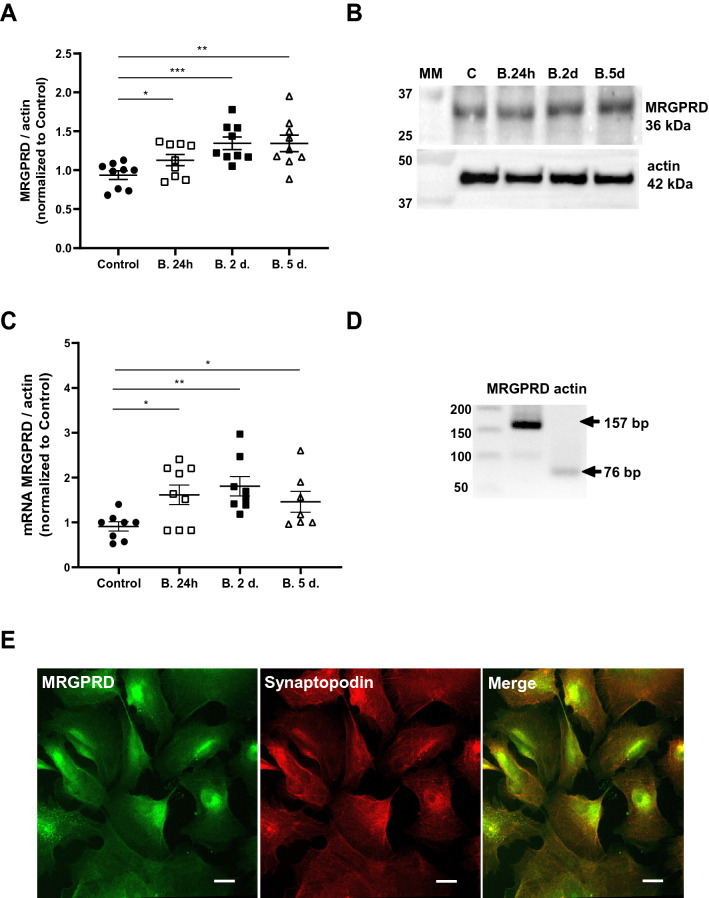
Human podocytes express extracellular L-BAIBA stimulation-sensitive MRGPRD. (**A**) MRGPRD protein expression in podocytes before and after L-BAIBA treatment (10 µM) for 24 h, 2 days, and 5 days. *n* = 9. *p < 0.05, **p < 0.01, ***p < 0.001. (**B**) Representative immunoblot membranes. (**C**) MRGPRD mRNA expression levels in podocytes before and after L-BAIBA treatment (10 µM) for 24 h, 2 days, and 5 days. *n* = 7–9. *p < 0.05, **p < 0.01. (**D**) Representative agarose gel with PCR products. (E) Confocal images of podocytes immunostained for MRGPRD and the podocyte marker synaptopodin. Scale bar = 10 μm.

We detected MRGPRD at the mRNA and protein levels in immortalized human podocytes (Fig. 1). The treatment of cells with L-BAIBA increased MRGPRD protein by 20% after 24 h and by ~ 44% after 2 and 5 days (Fig. 1A, B). MRGPRD mRNA levels significantly increased after the addition of L-BAIBA by 177% (24 h), 198% (2 days), and 160% (5 days; Fig. 1C, D). Confocal images of podocytes that were stained for MRGPRD and synaptopodin (a podocyte marker) showed intracellular localization of MRGPRD, which can be detected in the whole cell with greater fluorescent signal in perinuclear regions (Fig. 1E).

### L-BAIBA increases basal oxygen consumption and improves mitochondrial respiratory efficiency in podocytes

**Figure 2:**
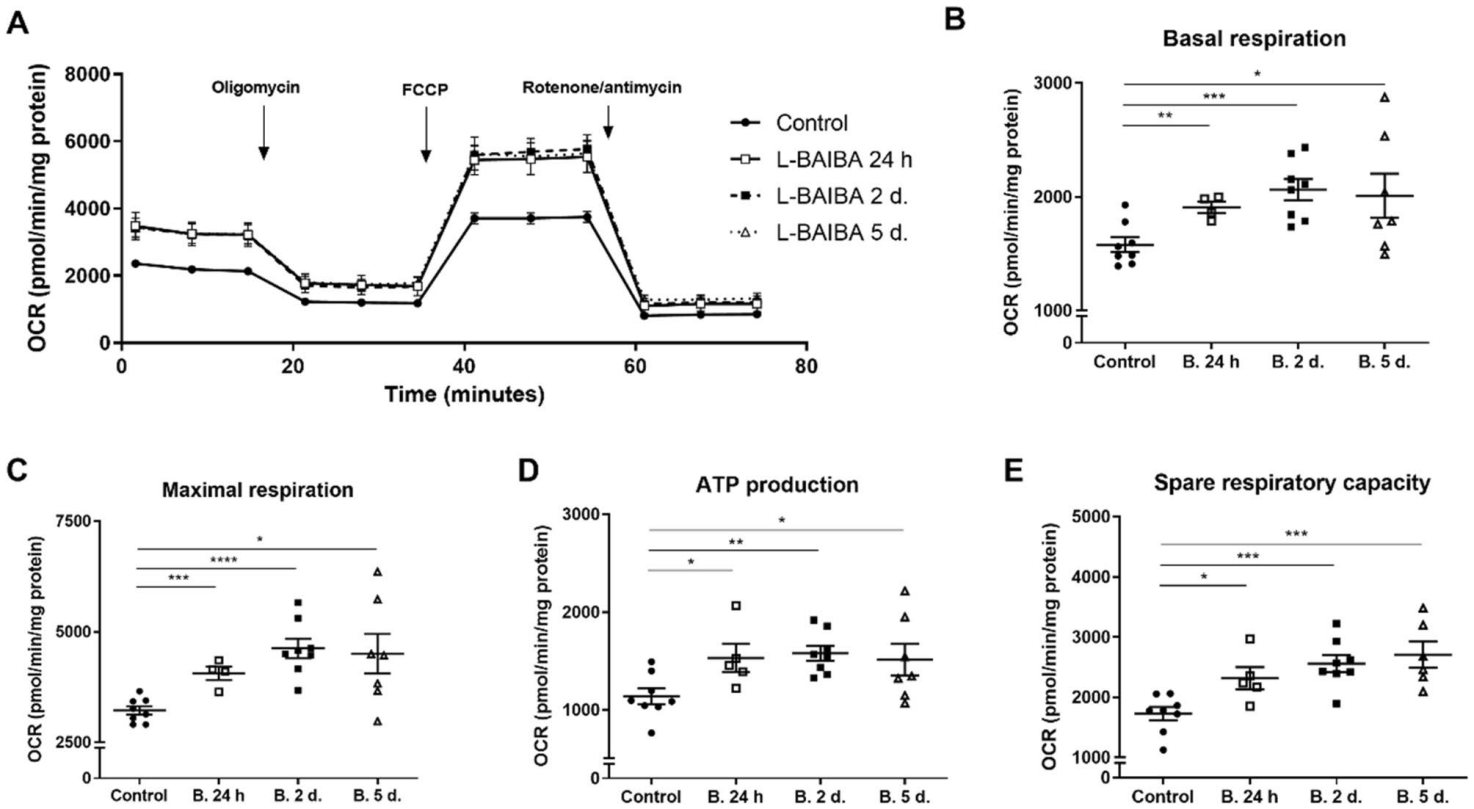
L-BAIBA improves podocyte respiratory efficiency. (**A**) Effects of L-BAIBA treatment (10 µM; 24 h, 2 days, and 5 days) on oxygen consumption rates (OCR) in podocytes after an injection of inhibitors of the respiratory chain (1 μM oligomycin, 1 μM FCCP, and 0.5 μM rotenone/antimycin). The data are expressed as mean ± SEM. (**B**) Basal respiration in podocytes in the presence and absence of L-BAIBA. (**C**) Maximal respiration in podocytes in the presence and absence of L-BAIBA. (**D**) Adenosine triphosphate (ATP) production in podocytes in the presence and absence of L-BAIBA. (**E**) Spare respiratory capacity in podocytes in the presence and absence of L-BAIBA. *n* = 4–8. *p < 0.05, **p < 0.01, ***p < 0.001, ****p < 0.0001.

We observed a profound effect of L-BAIBA supplementation on podocyte respiratory efficiency. L-BAIBA treatment increased overall OCRs (Fig. 2A), considerably enhanced basal and maximal respiration (basal respiration increased up to ~ 30% after 2 and 5 days of treatment; Fig. 2B), and increased maximal respiration up to 40–44% after 2 and 5 days (Fig. 2C). L-BAIBA induced a ~ 35% increase in ATP production in podocytes at 24 h, whereas longer incubation of podocytes with L-BAIBA did not result in further ATP production (Fig. 2D). Spare respiratory capacity time-dependently increased after L-BAIBA treatment (34% increase after 24 h, 48% increase after 2 days, and 57% increase after 5 days; Fig. 2E).

### L-BAIBA affects mitochondrial quantity and shape in podocytes

**Figure 3:**
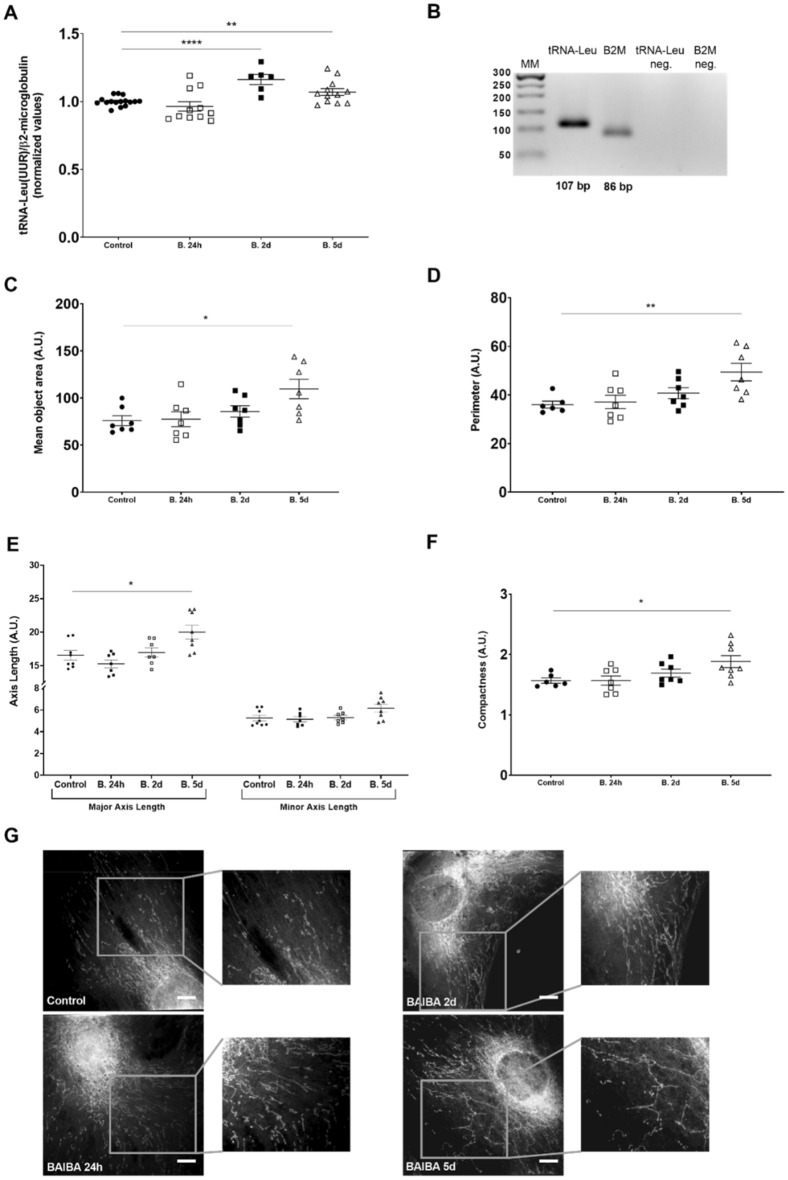
L-BAIBA promotes mitochondrial enlargement and branching in podocytes. (**A**) L-BAIBA treatment (10 µM; 24 h, 2 days, and 5 days) influenced mtDNA levels in podocytes. *n* = 6–16. **p < 0.01, ****p < 0.0001. (**B**) Representative agarose gel showing PCR products. (**C**) Mean mitochondrial area (total number of pixels in the object) in podocytes after L-BAIBA treatment. *n* = 7. *p < 0.05. (**D**) Mitochondrial perimeter (number of pixels that formed the boundary of an object) in podocytes after L-BAIBA treatment. *n* = 6–7. **p < 0.01. (**E**) L-BAIBA affected major axis length (pixel distance between endpoints on the longest line that could be drawn through the object) and minor axis length (longest line that could be drawn through the object while remaining perpendicular to the major axis) of mitochondria in podocytes. *n* = 7–8. *p < 0.05. (**F**) L-BAIBA affected mitochondrial compactness ([perimeter]^2^ / [4π × area]). *n* = 6–8. *p < 0.05. (**G**) Confocal images of podocyte mitochondria after L-BAIBA treatment. Scale bar = 10 μm.

The respiratory efficiency of podocytes is tightly related to mitochondrial dynamics. We investigated possible effects of L-BAIBA treatment on mitochondrial quantity by comparing ratios of mitochondrial DNA (mtDNA; tRNA-Leu) to nuclear DNA (nucDNA; β2-microglobulin) in podocytes before and after L-BAIBA supplementation (Fig. 3). We observed a ~ 15% increase in the mtDNA/nucDNA ratio after 2 days of treatment and a ~ 7% increase after 5 days (Fig. 3A, B). Interestingly, statistically relevant effects of L-BAIBA on mitochondrial size and shape were observed only after 5 days of extracellular L-BAIBA supplementation.

The mitochondrial area and perimeter (measured as the number of pixels that formed the boundary of each mitochondrion) increased by up to 44% and 37%, respectively, after 5 days of L-BAIBA treatment (Fig. 3C, D). The mitochondrial major axis length extended by 21% after 5 days of L-BAIBA treatment (Fig. 3E). Mitochondrial compactness increased by 20% after 5 days of L-BAIBA treatment, indicating a more irregular mitochondrial shape (i.e., elongation and branching; Fig. 3F).

### L-BAIBA promotes mitochondrial biogenesis by increasing PGC-1α and TFAM expression

**Figure 4:**
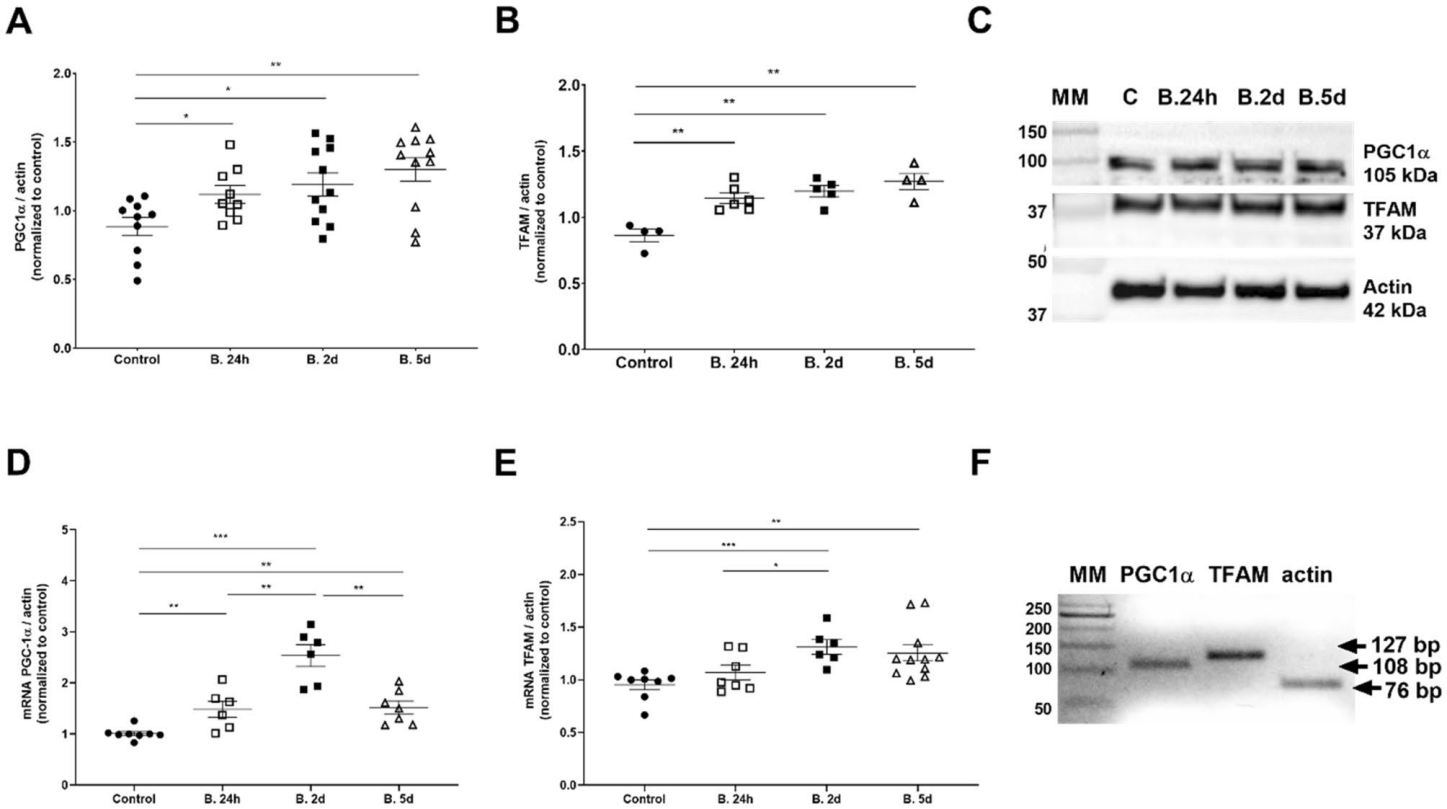
Mitochondrial biogenesis is enhanced in human podocytes after L-BAIBA treatment. (**A**) PGC-1α expression in podocytes that were cultured with and without L-BAIBA (10 µM; 24 h, 2 days, and 5 days). *n* = 9–11, *p < 0.05, **p < 0.01. (**B**) TFAM expression in podocytes after L-BAIBA treatment. *n* = 4–6. **p < 0.01. (**C**) Representative immunoblots for PGC-1α and TFAM. (**D**) PGC-1α mRNA expression in podocytes that were cultured with and without L-BAIBA. *n* = 6–8. **p < 0.01, ***p < 0.001. (**E**) Effects of L-BAIBA on TFAM mRNA expression in podocytes. *n* = 6–11. *p < 0.05, **p < 0.01, ***p < 0.001. (**F**) Representative agarose gel showing PCR products.

To elucidate whether the increases in mtDNA levels and elongation/branching of mitochondria in podocytes that were treated with L-BAIBA are related to an increase in mitochondrial biogenesis, we analyzed the expression levels of two markers of biogenesis, PGC-1α and TFAM, in cells that were cultured with L-BAIBA (10 µM; 24 h, 2 days, and 5 days). L-BAIBA significantly increased the expression of both PGC-1α and TFAM at the mRNA and protein levels, and its effects were largely time-dependent (Fig. 4). Protein levels of PGC-1α increased by 26%, 35%, and 47% after 24 h, 2 days, and 5 days of L-BAIBA treatment, respectively (Fig. 4A, C). Similarly, TFAM protein levels increased by 32%, 39%, and 47% in podocytes that were cultured with L-BAIBA (Fig. 4B, C). PGC-1α mRNA levels increased by ~ 50% after 24 h and 5 days of L-BAIBA treatment, whereas we observed a 2.5-fold increase in PGC-1α mRNA levels after 2 days (Fig. 4D, F). The effects of L-BAIBA on TFAM mRNA levels were seen mostly after 2 and 5 days of treatment (38% and 32% increases, respectively; Fig. 4E, F).

## Discussion

**Figure 5:**
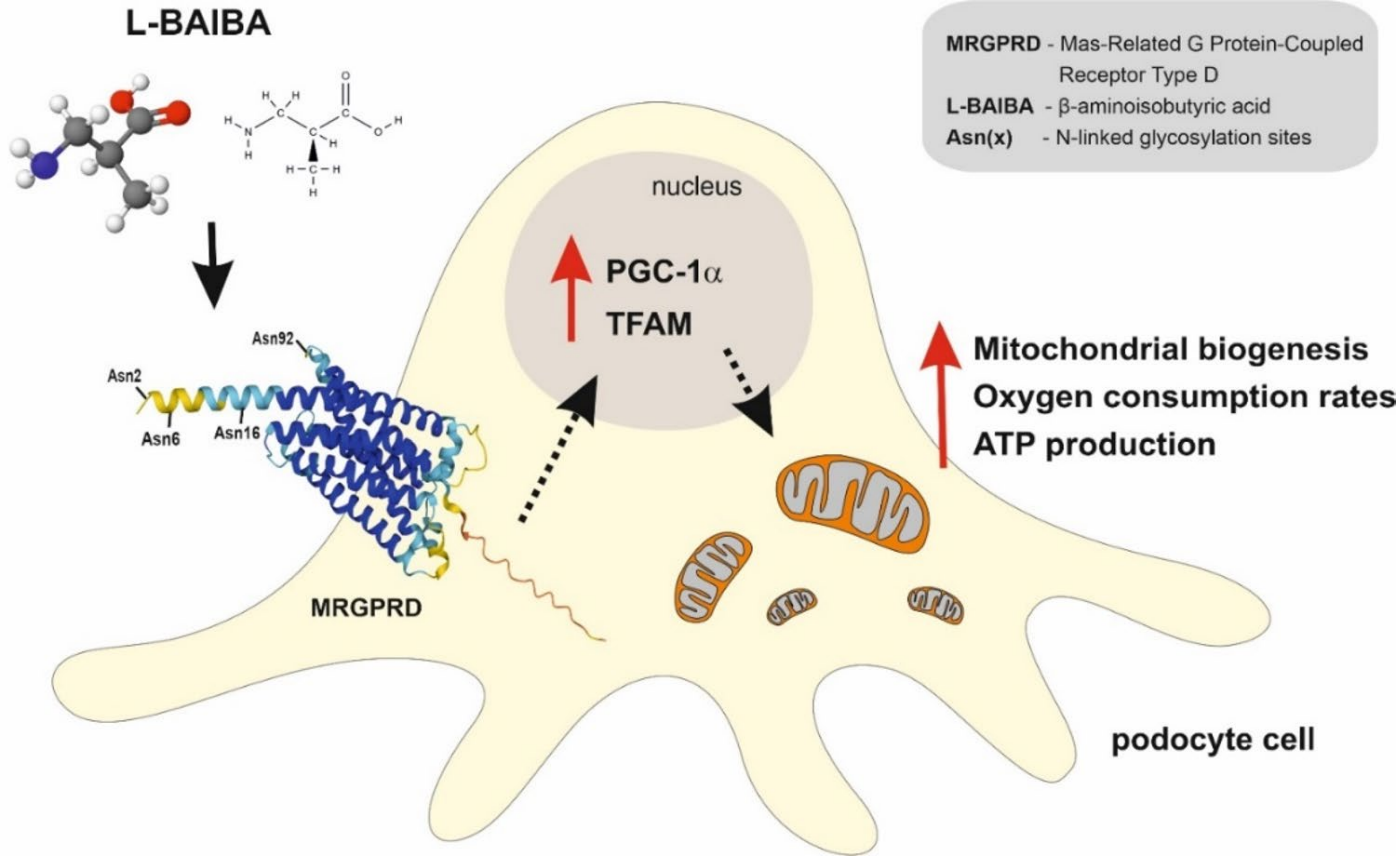
Schematic representation of potential mechanism of L-BAIBA action on mitochondria and respiratory functions in podocytes.

Elevated levels of plasma branched chain amino acids (e.g., valine, leucine, and isoleucine) have been linked with obesity, type 2 diabetes, and cancer^7,17,18^. L-valine is a precursor of L-BAIBA, which has beneficial effects on carbohydrate and lipid metabolism, mainly by enhancing insulin sensitivity, stimulating free fatty acid oxidation, and reducing proinflammatory cytokines^19^. The majority of studies of biological functions of BAIBA have been performed using racemic D/L-BAIBA, thus preventing the ability to distinguish specific effects of each enantiomer alone. However, evidence shows that L-BAIBA and not D-BAIBA is excreted by contracted muscle and binds to MRGPRD^15^. Our results showed that human podocytes express MRGPRD, the expression of which increased upon extracellular L-BAIBA supplementation. MRGPRD belongs to Mas-related G protein-coupled receptors and has been mostly associated with neuronal excitability and the sensation of pain and itch^20^. MRGPRD was first discovered in sensory neurons of the mouse dorsal root ganglion. Its expression was subsequently found in other organs and tissues, including arteries, testes, the urinary bladder, adipose tissue, and the gastrointestinal tract^21^. Although β-alanine is the best known MRGPRD ligand, it can also be stimulated by other molecules, such as alamandine^22^, angiotensin II^20^, and L-BAIBA^15^. The downstream effects of MRGPRD activation are multidirectional. In neurons, the stimulation of MRGPRD by β-alanine results in glutamate release and the attenuation of mast cell hyperresponsiveness and skin inflammation^23^. Alamandine-activated MRGPRD induced nitric oxide production in rats and thus may control vasodilation and fibrosis in the heart^24^. Evidence shows that BAIBA signaling through MRGPRD prevents reactive oxygen species-dependent mitochondrial breakdown in osteocytes^15^. Moreover, BAIBA treatment increased mitochondrial biogenesis in vascular endothelial cells^13^. We investigated whether BAIBA also plays a role in podocyte bioenergetics. Our results confirmed that L-BAIBA upregulated oxygen consumption, respiratory parameters, and ATP production in podocytes. We also found that L-BAIBA influenced mitochondrial size and shape in podocytes, inducing organelle elongation and branching, which likely promotes their respiratory efficiency. L-BAIBA strongly increased levels of the mitochondrial biogenesis markers PGC-1α and TFAM in podocytes, but the effect of L-BAIBA on mtDNA levels was only moderate, reaching a peak (115% of control) after 2 days of treatment. Mitochondrial biogenesis and dynamics are impaired in various renal pathologies, such as diabetic nephropathy^25^, focal segmental glomerulosclerosis^26^, and acute kidney injury^27^. Our previous findings showed that mitochondrial biogenesis and mitophagy were inhibited in podocytes that were cultured in a hyperglycemic environment and glomeruli that were isolated from rat models of diabetes^11^. We observed impairments in respiratory parameters in these podocytes and glomeruli, which can result from the dysfunction of mitochondrial dynamics. Interestingly, we found that the glomerular level of MRGPRD was reduced almost two fold in streptozotocin-induced (STZ) diabetic rats (supplementary Fig. S3A, B), indicating possible mechanism of downregulated BAIBA signaling and bioenergetic disturbance in diabetic kidney. Some attempts have been made to improve renal function by upregulating mitochondrial biogenesis in podocytes, but such studies have led to contradictory results. The podocyte-specific overexpression of a functional regulator of PGC-1α, lncRNA taurine-upregulated gene 1 (Tug1), rescued the expression of PGC-1α and improved mitochondrial bioenergetics in podocytes in diabetic mice^28^. Conversely, another study found that the podocyte-specific overexpression of PGC-1α led to the development of a collapsing glomerulopathy phenotype in mice, characterized by podocyte proliferation and dedifferentiation, proteinuria, and renal failure, likely attributable to excessive levels of PGC-1α^29^. Our findings indicate that L-BAIBA-induced PGC-1α upregulation has beneficial effects on podocyte bioenergetics, but further research is needed to determine whether L-BAIBA improves or reverses detrimental changes in podocyte mitochondrial metabolism and kidney function in disease states (Fig. 5).

## Supplementary Information

Supplementary Information.

## Data Availability

The datasets generated during the current study are available from the corresponding author on reasonable request.
