# Supplementary material for: β-Aminoisobutyric acid (L-BAIBA) is a novel regulator of mitochondrial biogenesis and respiratory function in human podocytes
Source: Sci Rep. 2023 Jan 14;13:766. doi: 10.1038/s41598-023-27914-8 (PMC9840613; doi:10.1038/s41598-023-27914-8)
Supplement: Supplementary file 1 — Supplementary Information. [file 41598_2023_27914_MOESM1_ESM.pdf]

Supplementary Information

Table S1. Primers and Taq-Man hydrolysis probes used in the experiments.

Gene	Accession no.	Primer sequence (5'-3')	Probe (5'-3')	Product
mtDNA tRNA^{Leu}	NC_012920.1	Forward: CACCCAAGAACAGGGTTTGT<br>Reverse: TGGCCATGGGTATGTTGTTA	—	107 bp
nDNA β2-microglobulin	NC_000015.10	Forward: TGCTGTCTCCATGTTTGATGTATCT<br>Reverse: TCTCTGCTCCCCACCTCTAAGT	—	86 bp
MRGPRD	NM_198923.2	Forward: CCGTGGAGTCAGCCCTAAAC<br>Reverse: CAGAAGGGGTTCCTGTGCAT	CTGCTGGG	157 bp
TFAM	NM_003201.2	Forward: GTTTCTCCGAAGCATGTGG<br>Reverse: AGATGAAAACCACCTCGGTAAA	TGCCCTGG	127 bp
PGC-1α	NM_001330751.1	Forward: CACCCTCTTCTCTTCCTTCTTTT<br>Reverse: GGGGCTCCAATTTTACCAAT	CCTCCTGG	108 bp
β-actin	X00351.1	Forward: ATTGGCAATGAGCGGTTC<br>Reverse: GGATGCCACAGGACTCCA	CTTCCAGC	76 bp

Table S2. Primary antibodies used in Western blot experiments.

Antibody	Clonality	Dilution	Source	Catalog no.
TFAM	monoclonal	1:200	Santa Cruz Biotechnology	sc-166965
PGC1α	monoclonal	1:125	Santa Cruz Biotechnology	sc-517380
MRGPRD	monoclonal	1:1000	Abcam	ab155099
Synaptopodin	polyclonal	1:50	Santa Cruz Biotechnology	sc-21537
Actin	monoclonal	1:10000	Sigma-Aldrich	A3853

Figure S1. Uncropped immunoblot membranes

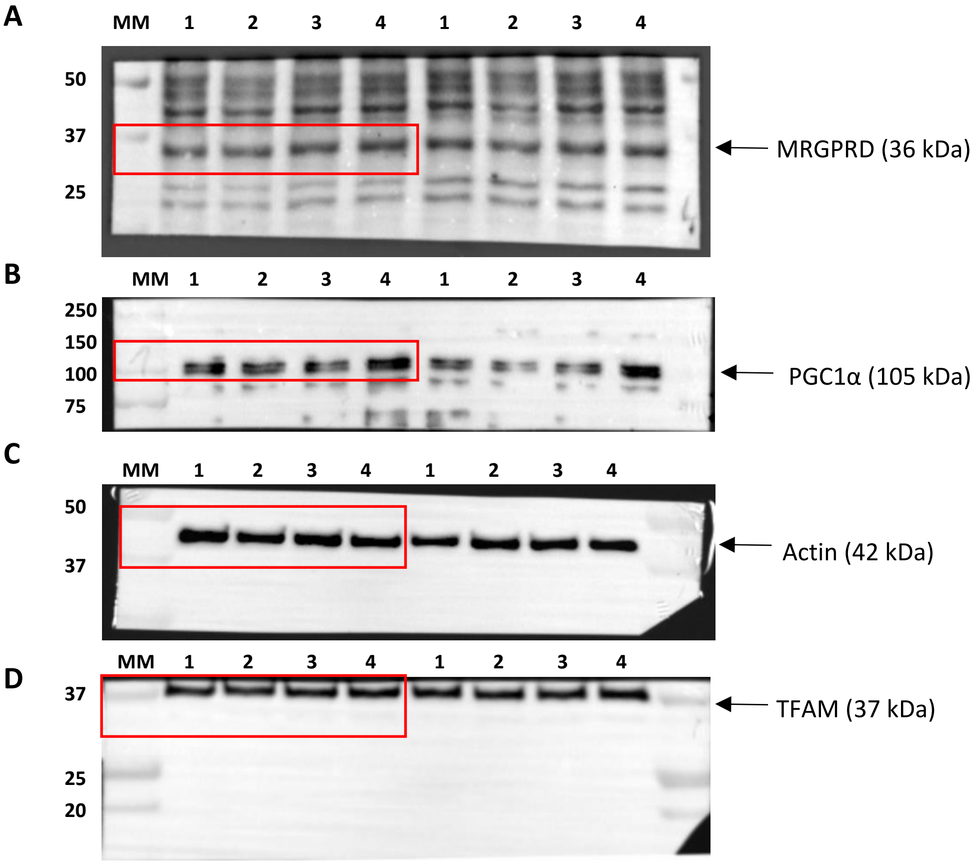

Fig. S1. Immunoblot membranes. A. Mas-related G protein-coupled receptor type D (MRGPRD) receptor. **Original for Fig. 1b**. B. Peroxisome proliferator activated receptor α coactivator-1α (PGC1α). **Original for Fig. 4c.** C. β-actin. **Original for Fig. 1b and 4c.** D. Transcription factor A mitochondrial (TFAM). **Original for Fig. 4c.** Lane 1 – control, 2 – BAIBA 24 h, 3 – BAIBA 2 days, 4 – BAIBA 5 days. MM – molecular marker.

Figure S2. Gels with PCR products

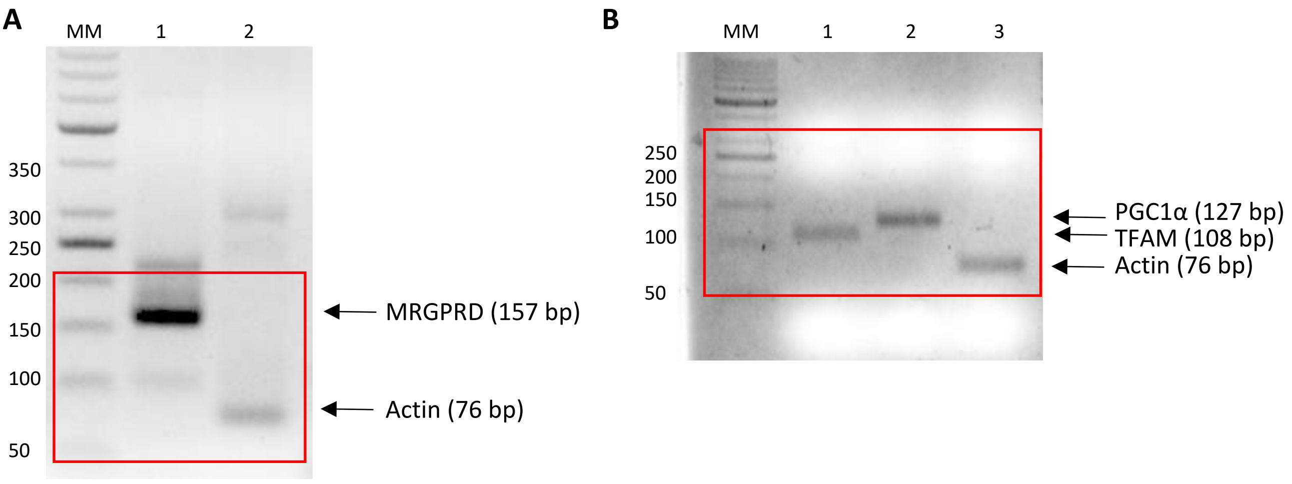

C

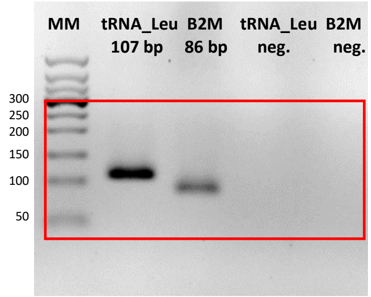

Fig. S2. Agarose gels 2.5% showing PCR products. A. MRGPRD receptor (157 bp; lane 1) , β-actin (76 bp; lane 2). **Original for 1d**. B. TFAM (108 bp; lane 1), PGC1α (127 bp; lane 2), β-actin (76 bp; lane 3). **Original for 4f.** C. DNA levels tRNA_Leu (mtDNA) (107 bp), β2-microglobulin (86 bp), negative controls. **Original for 3b.** MM – molecular marker.

Figure S3. MRGPRD levels in glomeruli isolated from diabetic rats.

The *in vivo* relevance of the observations from podocyte cell culture was verified using glomeruli isolated from rat model of diabetes. In streptozotocin-induced (STZ) diabetic rats, which were characterized by hyperglycemia and overt proteinuria, we observed that glomerular level of BAIBA receptor MRGPRD was reduced to ~54% of the control (Fig. S3A, B).

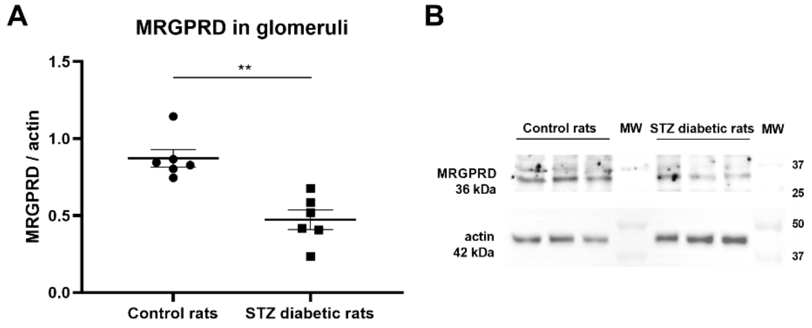

Fig. S3. BAIBA receptor, MRGPRD, is decreased in glomeruli isolated from streptozotocin-induced (STZ) diabetic rats. A. MRGPRD protein levels in freshly isolated glomeruli from control and diabetic rats. N = 6, ***p* < 0.01. B. Representative immunoblots (1,2,3 – different rats).

Immunoblot membranes for quantification of PGC1α and TFAM.

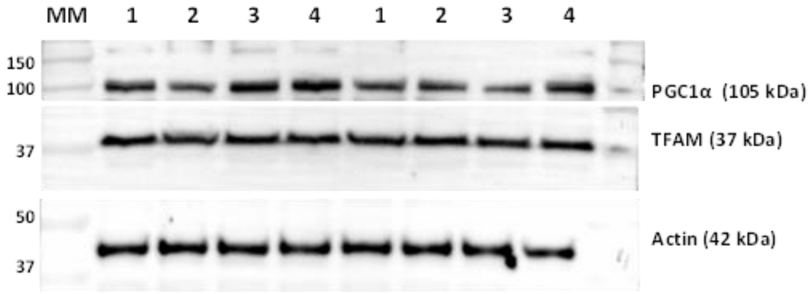

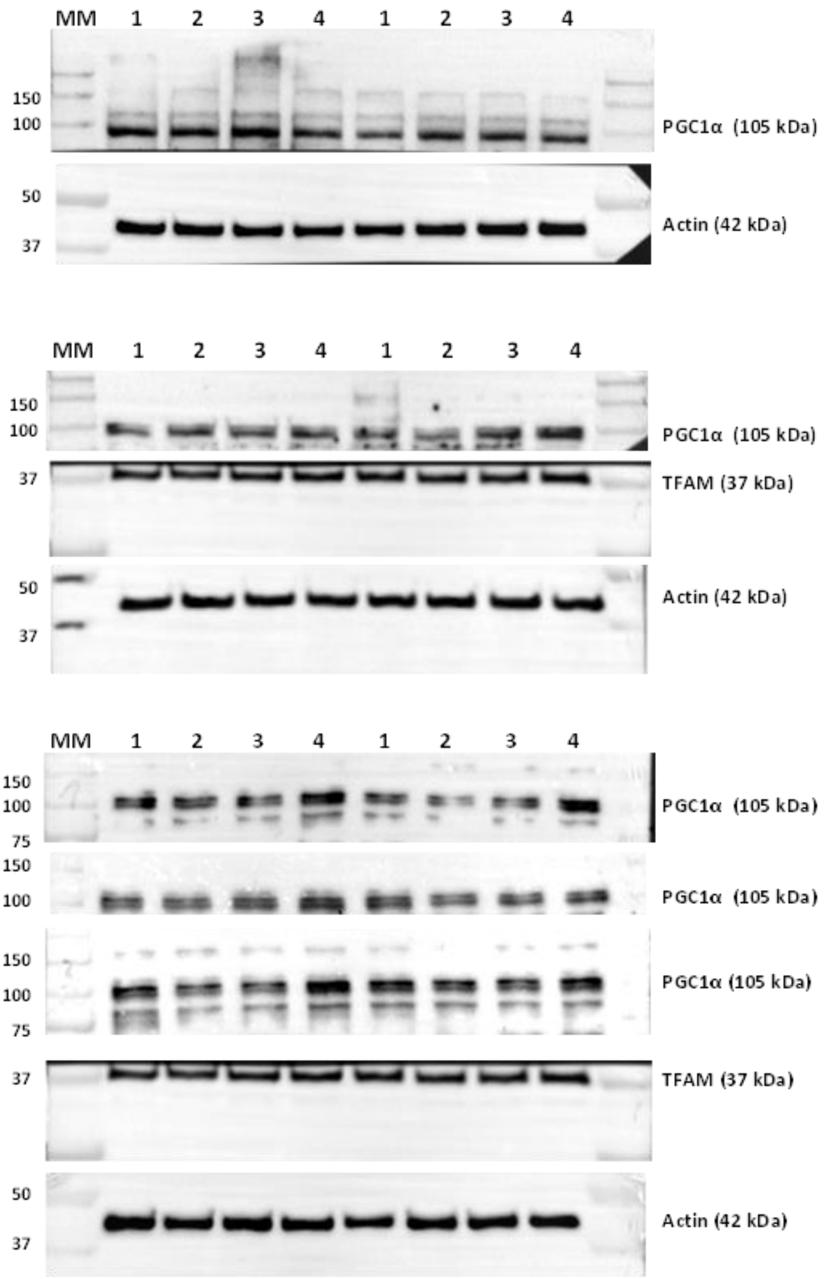

Immunoblot membranes. Lane 1 – control, 2 – BAIBA 24 h, 3 – BAIBA 2 days, 4 – BAIBA 5 days. MM – molecular marker.
